# Systematically Prioritizing Candidates in Genome-Based Drug Repurposing

**DOI:** 10.1089/adt.2019.950

**Published:** 2019-12-13

**Authors:** Anup P. Challa, Robert R. Lavieri, Judith T. Lewis, Nicole M. Zaleski, Jana K. Shirey-Rice, Paul A. Harris, David M. Aronoff, Jill M. Pulley

**Affiliations:** ^1^ Vanderbilt Institute for Clinical and Translational Research, Vanderbilt University Medical Center, Nashville, Tennessee.; ^2^ Department of Biomedical Informatics, Vanderbilt University Medical Center, Nashville, Tennessee.; ^3^ Division of Infectious Diseases, Department of Medicine, Vanderbilt University School of Medicine, Nashville, Tennessee.; ^4^ Department of Pathology, Microbiology and Immunology, Vanderbilt University School of Medicine, Nashville, Tennessee.

**Keywords:** drug repurposing, genomics, phenome-wide association study (PheWAS), precision medicine, informatics

## Abstract

Drug repurposing is the application of approved drugs to treat diseases separate and distinct from their original indications. Herein, we define the scope of all practical precision drug repurposing using DrugBank, a publicly available database of pharmacological agents, and BioVU, a large, de-identified DNA repository linked to longitudinal electronic health records at Vanderbilt University Medical Center. We present a method of repurposing candidate prioritization through integration of pharmacodynamic and marketing variables from DrugBank with quality control thresholds for genomic data derived from the DNA samples within BioVU. Through the synergy of delineated “target-action pairs,” along with target genomics, we identify ∼230 “pairs” that represent all practical opportunities for genomic drug repurposing. From this analysis, we present a pipeline of 14 repurposing candidates across 7 disease areas that link to our repurposability platform and present high potential for randomized controlled trial startup in upcoming months.

## Introduction

Drug repurposing is the practice of developing approved drugs for uses in new, unexplored indications.^1^ Repurposing initiatives have seen major growth in recent years, partially energized by the industrial undertaking of repurposing projects in parallel to the development of new pharmaceuticals. There are numerous benefits of this approach to drug development that are of interest to the pharmaceutical industry. Repurposing is an efficient approach to drug development, as it makes use of existing knowledge on pharmaceuticals and their mechanism(s) of action, thus minimizing the need for much of the preclinical and early safety work required by new chemical entity (NCE) drug discovery and development. Many bioinformaticians believe that drug repurposing is one potential solution to Eroom's Law: the observation that the efficiency of drug development halves about every decade.^2^ Thus, repurposing may offer a systematic approach to increasing the pace of therapeutic development. One of the benefits of drug repurposing is cost reduction; recent estimates suggest that marketing approval through repurposing can cost ∼70% less than that for NCE drug development projects. Drug repurposing is often much faster than NCE drug development: although the time from discovery to regulatory approval of an NCE may require 12 years or longer, repurposing can establish the same task in <6 years, nearly half the conventional time.^3^

Of recent interest, several large pharmaceutical companies, including Teva Pharmaceutical Industries, Ltd, have sought partnerships with academic bioinformatics centers and data science entities. This has manifested in machine learning partnerships—as with Teva and IBM Watson—through which Teva has made use of Watson's natural language processing abilities to digest unstructured data in electronic health records (EHRs).^4^ Within academia, there is a similar focus on creating industrial relationships, through which academic biobanks partner with pharmaceutical companies to help fuel the development of both repurposable drugs and NCEs.^5^ On the contrary, pharmaceutical partners are eager consumers of research innovations and can provide the industrial machinery required for the reformulation of repurposing candidates, as repurposed drugs can be re-engineered for maximal safety and deliverability before they are included in phase I/II trials.^6^

An issue omnipresent in NCE drug discovery is “off-target” (*i.e.*, of nonintended targets) compound effects, specifically when these effects are likely to pose safety concerns or reduce drug efficacy. Although “on-target” toxicity is also a legitimate concern, this issue is more readily addressable, because drug development teams are likely to study the biology of an intended target fully. Hence, “off-target” effects are concerning, as genomic data solely address target biology: genomic data give no information specific to a given drug or molecule's “off-target” effects. With this information, it may be possible to predict the “on-target” toxicity of a molecule of interest—for known target(s)—although genomic data provide no frame of reference for targets that are otherwise unknown. In drug repurposing, however, existing clinical data (about exposure to the drug itself) mitigate the risk of uncovered “off-target” effects: because all potential repurposing candidates have been dosed extensively in humans, it is much less likely—compared with NCE drug discovery—that unexpected “off-target” toxicity issues will emerge in a repurposing project. At present, the failure of most drugs is attributable to lack of efficacy (rather than lack of safety or unacceptable pharmacokinetics); however, it is still true that preclinical safety data are not sufficient to accurately predict the risk of clinical failure from safety issues.^7^ Fortunately, extensive human experience mitigates this safety failure risk for approved drugs, and efficacy failure risk is reduced through the application of human genomic data.

Thus, with the concurrent demands of drug repurposing and personalized medicine, precision drug repurposing is becoming increasingly relevant. The simultaneous analysis of drug databases and genomic data allows for the development of precision repurposing schemes capable of detecting new indications for drugs selected on the basis of human genomic evidence, implicating their targets with specific diseases. Nonetheless, given the large number of available drugs—and the vastly larger space of human genomic information—new methods are required to systematically screen molecules on the pharmacologic and genomic basis of their repurposability.

In this study, we propose an algorithmic scheme for the selection of repurposing candidates using relevant drug profiles from DrugBank,^8–12^ a public database of compounds that have at least been in a phase I clinical trial, and genomic data from BioVU, a biobank of patient samples from Vanderbilt University Medical Center (VUMC).^13,14^ This unbiased, systematic, data-driven approach has allowed us to define the “repurposable drugged genome,” stemming from the intersection of the “drugged genome” and targets for which we have high-quality genomic data, as described hereunder.

### DrugBank

DrugBank 5.1.2 (updated December 20, 2018) is a publicly accessible drug referencing tool developed by the University of Alberta; it contains encyclopedia-like entries on common pharmaceutical agents, including >200 data fields on each drug entry (“DrugCard”). As a self-described bioinformatics and cheminformatics resource, DrugBank lends itself to multivariate characterization of drug form and function, providing a highly user-friendly application programming interface (API) toward the extraction of relevant data fields.^15^

The most pertinent data sets for this investigation involve DrugBank genomics, described on DrugCards as the associated genes for each drug target. This information facilitates the overlap of drug metrics and BioVU single-nucleotide polymorphism (SNP)/single nucleotide variant (SNV) data through linkage of target genomics; in turn, drug repurposability is evaluable by the minor allele frequency (MAF) and genotype call quality of SNPs/SNVs on drug target genes. This information is then mappable to new indications in the evaluation of phenome-wide association study (PheWAS) data.

For the aforementioned reasons, DrugBank was selected as the source of all “drugged genome” candidates for this investigation. This database was also selected for its regular update schedule (daily DrugCard information uploads, biennial database-wide refreshments^9^), breadth of potential repurposing candidates, and flexible API.

### BioVU

BioVU is a repository of DNA samples extracted from excess blood samples in the clinical testing of both adult and pediatric patients at VUMC. The major hallmark of this biobank is its linkage to longitudinal, de-identified EHRs^16^ (Synthetic Derivative^17^): samples remain linked to the de-identified medical records of the patients from whom they were collected. The result is a research-ready data set, with patient DNA and genomic data continuously linked to health record information.^16^

In this study, we leverage BioVU to perform PheWAS,^18^ testing for associations between SNPs/SNVs within drug target genes, and clinical phenotypes defined by billing codes. Thus, PheWAS is able to map phenotypes to associated genomic alterations in drug target genes.^19,20^

This study includes the integration of SNP/SNV data from an Illumina Infinium HumanExome BeadChip array (hereafter referred to as “ExomeChip”) genotyping platform^21^ in BioVU with the known “drugged genome” from DrugBank. With this synthesis, pharmacodynamic (PD) and genomic attrition algorithms—as defined hereunder and in the Supplementary Data S1 of this article—are applied across both data sets, producing “shortlists” of candidates for drug repurposing. Subsequently, PheWAS call quality for prioritized drug-SNP/SNV pairs is analyzed as previously described^20^ to support a growing pipeline of drug repurposing projects at VUMC.

## Methods

Given there are >2,000 approved drugs (inclusive of 3 international regulatory agencies) and >11,000 compounds through phase I clinical trials, new methods to improve screening of available compounds are essential to developing new drug repurposing projects. We propose in this report a repurposing prioritization scheme based on candidate targets and mechanisms of action, such that candidates are ranked pragmatically for the launch of new drug repurposing projects.

Thus, the core of this investigation involved our ability to develop an efficient attrition workflow, incorporating VUMC's BioVU/PheWAS data and DrugCards from the mined database of potential candidates.

To extract the pharmacological data necessary for this repurposing project, the complete DrugBank database was downloaded from the repository website (https://www.drugbank.ca/releases/latest). As of November 2017 (update 5.1.0), this database included information on 10,505 drugs, stored in eXtensible Markup Language (XML) format. In the DrugBank XML database, each drug has one record with >1,700 descriptive lines.

The following fields were extracted for each drug using the R programming language^22^ and its XML and plyr packages to scope the entirety of the XML database:

- name
- type (“biotech” or “small molecule”)
- status (“approved,” “illicit,” “investigational,” “vet-approved,” “nutraceutical,” “withdrawn”)
- number of targets
- number of targets with known action (“known-action = = ‘yes’”)
- For each target with known action:
  - target name
  - action
  - gene name
- number of enzymes
- list of enzymes
- number of transporters
- list of transporters

Following application of PD and genomic screens relevant to an evaluation of potential repurposing, we extracted data on earliest marketing start date and country of approval for each molecule on a repurposing “shortlist.” Country of approval information was scraped for completeness of the approval status data set, and marketing data were extracted as a proxy for potential intellectual property opportunities. Again using the XML package in R, details related to the “products” variable were extracted from the database for each of drug “country” and “started-marketing-on.” A custom function in R, *getMarketingDetails*, was executed to create a spreadsheet with these marketing details for any desired list of drugs. The R code used to extract relevant information for this drug repurposing study is available in the GitHub repository (https://github.com/judytlewis/drugRepurposing).

Thus, the entirety of the DrugBank database was first extracted as an XML file using the statistical software R, giving 10,505 potential repurposing candidates (*n* = 10,505). The following variables were then considered:

1. drugName (the listed name of each drug)
2. type (a binary categorization of drug type: small molecule or biologic)
3. status (official categorization by U.S. Food and Drug Administration [FDA] and/or Health Canada (*e.g.*, approved, approved/investigational))^23^
4. countryApproved (United States and/or Canada)^23^
5. marketingStartDate (date of first marketing, in United States and/or Canada)^23^
6. numberOfTargets (number of known targets for each candidate)
7. numberOfTargetsWithKnownAction (number of known targets with further known pharamacological mechanism for each candidate)
8. target *(i)* (listed target for each candidate, with *n* representing iteration per known target in numberOfTargets (*i* (*i* ∈ [1,25]))
9. action (*j)* (listed action (*e.g.*, inhibitor, activator) for each candidate, with *j* representing iteration per known target in numberOfTargets (*j* ∈ [1,25]))
10. geneName (*k)* (associated gene for each target in numberOfTargets, iterated *k* times (*k* ∈ [1,25]))

Controlled analysis of each of these parameters allowed for efficient attrition, by which systematic consideration of PD for each candidate was used as a basis for stepwise parsing. Thus, the following screens were applied, given the task of defining the “repurposable drugged genome.”

### Drugs by Type

Given the significant cost difference of obtaining small molecules for clinical trials, as compared with biologics,^24^ and the binary nature of drug type classification,^24^ separation of mined agents as “biologics” and “small molecules” quickly identified drugs more easily accessible (*i.e.*, small molecules) from those generally less accessible (*i.e.*, biologics). Our list is intended to be both computationally valid and pragmatic in its application to high-throughput screening of identified drug repurposing candidates. Therefore, biologics were excluded after this first round of analysis, given the practical difficulties in obtaining many of these agents.^25–27^ This discrepancy between small molecules and biologics is easily illustrated by comparing the small molecule misoprostol^28,29^ to biologics that would also make sense to repurpose for a similar new use.

Namely, misoprostol is a prostaglandin-derived small molecule currently approved for the treatment of iatrogenic ulcers, resulting from overuse of nonsteroidal anti-inflammatory drugs (NSAIDs).^28^ An ongoing randomized, double-blind, placebo-controlled, phase II clinical trial (NCT03617172) led by Dr. David Aronoff from VUMC is testing the repurposability of misoprostol for the prevention of recurrent *Clostridioides difficile* infection, the leading cause of antibiotic-associated diarrhea.^28^ Per generalized DrugBank market data, the median price per tablet of misoprostol is $2.33.^30^ In contrast, the cost of obtaining a biologic agent similarly indicated for gastritis (*e.g.*, adalimumab^26^) prohibits purchasing large amounts of the biologic necessary to conduct a clinical trial. A review of market data for adalimumab gives the average cost per dose in the United States to be $2,669,^31^ roughly 1000-fold greater than the average price per tablet of misprostol. Similarly, a recent study estimates the price of bezlotoxumab, a biologic agent for preventing recurrent *C. difficile* infection, as $4,560 per vial.^32^ Given that biologics are often proprietary, although many established small molecules are off-patent, the issue of limited drug access plagues repurposing studies of biologics. Removing all biologics left 9,292 potential repurposing candidates available for review from the total listing of 10,505 drugs.

### Drugs by Approval Status

A major aim of repurposing is the development of new therapeutic strategies among sets of agents currently in use for a wide variety of indications. For repurposing to remain practical within the academic medical center setting and within a reasonable timeframe, repurposing candidates must be approved for clinical use, or at least through a phase I clinical trial.

The data in DrugBank include approval status for each cataloged agent in keeping with the labels established by the FDA, European Medicines Agency, and Health Canada.^23^ Therefore, only drugs with a listing “Approved” were considered as repurposing candidates. Specifically, drugs with the labels provided in *Appendix Table A1* were retained for further consideration.

Attrition by approval status left 2,219 potential repurposing candidates from the total listing of 10,505 investigational drugs.

### Drugs by Number of Targets of Known Mechanisms of Action

Noting the focus of drug repurposing methods on “selective” drugs (ideally, drugs of one target of known mechanism of action [MOA]), we decided to consider only drugs with one known target and MOA. Although future studies may consider more complex pharmacology (*i.e.*, drugs with multiple known targets [and thereby multiple gene targets for analysis]), the preliminary nature of this scan dictated restriction to drugs of one target with known MOA.

Parsing by targets of known MOA left 823 potential repurposing candidates from the original listing of 10,505 investigational drugs.

#### Exclusions

Based on existing knowledge of drug toxicity, drugs of limited potential owing to significant safety issues were manually removed from the agent shortlist, in consultation with pharmacologists. Our repurposing method relies on human genomic data^20^; thus, we focus solely on drugs with human protein targets. Hence, drugs with nonhuman targets (fungal, viral, helminthic, and bacterial nucleic acids) were removed from this data set. Given that many anticancer agents work by damaging DNA (*e.g.*, alkylating agents), these drugs were also excluded from consideration. Drug entries with missing or null entries in any aforementioned data field were additionally parsed.

#### Consolidations

Given that drugs of the same class (*e.g.*, ACE inhibitors) are inherently redundant in affecting the same target, we decided to move from a per-drug attrition scheme to a per-*drug class* parsing strategy. To accomplish this task, “target-action pairs” were defined through the simultaneous association of each drug target and its DrugBank-specified action (*e.g.*, inhibitor, activator). Thus, all drugs were grouped into “target-action pairs” and handled in this manner for the remainder of the study.

After exclusion and consolidations, 621 remaining small molecules were grouped into 237 unique “target-action pairs.” MAF data—a cutoff of 0.1% generally means we have sufficient data to run the PheWAS analysis and observe meaningful/new associations for a given drug target—were then integrated into the list of agents to reach a working, precision model.

### Merger with Genomic Data

First, a comprehensive list of SNPs/SNVs on the exome chip^21^ (see BioVU section)—along with their unique ExomeChip ID numbers (exmID), reference cluster ID numbers (rsID),^33^ gene name (Gene), MAF, mutation type and listing (Mutation), major base pair (A1), and minor base pair (A2)—were mined from the Chip gene annotation file and BioVU databank of genotypes to an XML file using the statistical software R. This gave 239,796 SNPs/SNVs available for review.

A selectivity screen was then applied to the mined SNP/SNV data: first, SNPs/SNVs with rsID listed as “NULL” were removed from consideration, given the inability to verify SNP/SNV information with established genomics databases, including dbSNP (https://www.ncbi.nlm.nih.gov/snp).^33^ For consistency in data handling, we considered only SNP/SNV querying by rsID; we acknowledge that this method does not allow for enrichment of our SNP/SNV data set outside dbSNP, by neglecting the possibility of discovering additional SNP/SNV information from another database. However, given that dbSNP information that we do not curate is most likely dominated by rare variants^34^—and our purging of rare variants from consideration, for practical reasons—we do not consider this limitation to be significant.

Next, SNPs/SNVs with genotyping missingness >0.05 and MAF <0.001 for white populations—the dominant demographic represented in the available PheWAS data—were removed from the ExomeChip. Given the implementation of PheWAS-based algorithms in this investigation, 0.1% frequency was used as “utility” benchmark, noting optimization of PheWAS performance at MAF >0.001 and establishment of this limit as an appropriate cutoff in previous literature.^35,36^ Indeed, using frequency conventions used by the National Center for Biotechnology Information,^35^ MAF values ≤0.001 are deemed “rare,” rather than “minor.”

Under an assumption of stochastic missingness,^37^ SNPs/SNVs with missingness >5% were parsed from the model to prevent confounding bias within phenotypic associations.^38^

Application of these screens gave 58,945 eligible SNPs/SNVs—tied to (de-identified) patient electronic medical records at VUMC—from the original listing of 237,796 variants.

Synthesis of the pharmacological data and narrowed ExomeChip data were now feasible, whereby shortlisted drugs were further reduced on coverage of target genes in our genomic data set. This was accomplished by application of the “target-action pairs” strategy, allowing genomic comparison between target-associated genes and eligible SNPs/SNVs. The number of “target-action pairs” with genes and eligible SNPs/SNVs cross-listed on our ExomeChip was then determined. Thus, it was determined that 227 “target-action pairs” of the pool of 237 “pairs” demonstrated cross-listed SNPs/SNVs, giving 96% total SNP/SNV coverage for the ExomeChip population in BioVU. These 227 distinct “target-action pairs” translate to 147 unique targets that may be further probed for repurposing potential.

**Appendix Fig. A1.**
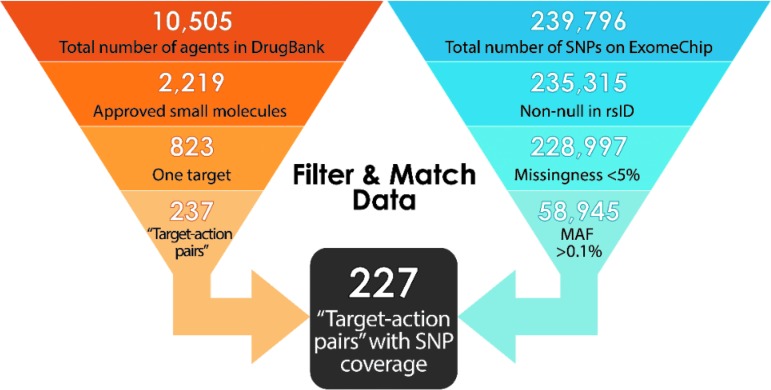
A summary representation of the pharmacodynamic and genomics-inspired workflow developed for the selection of optimal repurposing candidates: attrition at each step is defined through genomic and chemical profiling of ideal small molecule candidates. MAF, minor allele frequency; rsID, reference cluster ID number; SNP, single-nucleotide polymorphism.

A representation of the holistic attrition strategy is given in *Appendix Figure A1*. Example “target-action” pairings are listed in *Appendix Table A2*. Furthermore, Supplementary Data S2 to this article is given, which may be accessed as “Supplement.md” through the GitHub repository at https://github.com/judytlewis/drugRepurposing. In this supplement, we provide listings of drugs and “target-action pairs” considered at each stage of the above-described filtration, and a listing of the 227 “target-action pairs” we believe to encompass all pragmatic opportunities in genome-based drug repurposing and their associated marketing information.

## Discussion of Results and Study Limitations

Our model effectively produces a prioritized set of drugs that are promising candidates for our specific method of repurposing, by reducing ∼11,000 drugs and ∼240,000 SNPs/SNVs to a prioritized set of 227 “target-action pairs,” given specific criteria on drug target number and approval status, along with SNP/SNV coverage and MAF.

Nonetheless, two conditions restrict our outputs, in that this model only operates on small molecules with one target of known MOA. Given that 518 agents of the 621 drugs with one target of known MOA have only one total target listed in DrugBank (*i.e.*, 83.41% one-target-total rate), the aforementioned statement may be simplified to a requirement of small molecules of no more than one total target. Clearly, drug specificity is a relative concept and a complete specificity/activity profile against all possible drug targets does not exist for any drug, much less *all* approved drugs. However, we have taken some obvious quality control steps, such as removing DNA damaging agents and drugs known to affect nonhuman targets from our data set.

The above assumptions were used both in consideration of the ideal characteristics for repurposing candidates, along with an understanding of this investigation as a preliminary attempt at the design of a repurposing candidate “search engine.” In short, this investigation aimed to develop a method by which the entirety of the “drugged genome” may be further narrowed to the “repurposable drugged genome.”

In addition, drug toxicity was not considered systematically in the attrition of potential repurposing candidates. Although candidates with severe and obvious toxicity concerns—as relevant to utility for repurposing—were manually removed, it was not feasible to mine structured toxicology data from DrugBank, given the absence of an absolute measure of toxicity in the field of pharmacology. In addition, safety issues were difficult to assess, as these remain relative to selected patient populations and besought indications.

On the whole, our method of repurposability screening produces a shortlist of 147 unique targets that may be considered further for drug repurposing. This result is derived through PD screening of all approved small molecules across established governmental regulatory organizations, along with genomic screening of all variants associated with their druggable targets. Hence, our filtration reflects pharmacological, genomic, and pragmatic considerations of drug repurposing. Indeed, one cannot understate the importance of real-world considerations in the selection of drug repurposing candidates. Even for a large, relatively well-funded drug repurposing program—such as that at VUMC—time and money are limiting factors, as the identification and phase II efficacy study of a repurposing candidate often requires $3–$8 million and several years of time. Therefore, it is only feasible for an academic medical center-based program to launch a handful (or fewer) of repurposing programs in a given year.

Our method of repurposability screening accommodates these limitations by providing a comprehensive database of all potential leads for genomic drug repurposing. This framework supports the upstart of new randomized controlled trials (RCTs), as all candidates for which genomic drug repurposing is possible are listed in a centralized location. In turn, repurposing programs using this resource may save significant drug discovery resources necessary for scoping the existing pharmacopeia for repurposing hits.

Our repurposing program has used a similar strategy in the generation of its repurposing pipeline. Thus, we present test cases of the above workflow across seven disease areas, for which we pull genomic repurposing shortlists from the output of systematic pharmacological and genomic analyses. Hence, our current pipeline of 14 repurposing candidates (including 3 drugs in ongoing RCTs [NCT03694249, NCT03617172, NCT03527472]) is derived from workflows analogous to those in this article; because all these molecules are in funded clinical trials, we conclude that the attrition method presented above is largely successful in supporting trial startup.

A summary of our current drug repurposing pipeline—as traceable to this study—is given in *Appendix Table A3*.

We also compare our findings with those presented in a recent scope of the genomic repurposing space by Finan *et al.*^39^ In this publication, Finan *et al.*^39^ present genome-wide association study (GWAS)-derived target-SNP pathogenicity relationships that have application in stimulating new drug repurposing projects. The authors assert that the druggable genome—defined by 4,479 genes corresponding to protein targets able to bind available large and/or small molecules^39,40^—may be reduced to a total of 144 drug repurposing targets, as identified by analysis of SNP pathogenicity in druggable genes within their GWAS data sets.^39^ Our analysis presents a similar statistic while focusing on the identification of repurposing candidates and considering drug-specific attrition criteria and target directionality. Thus, we count 147 unique targets that are eligible candidates for repurposing. Our methods contain pharmacological screening of potential repurposing candidates, identifying repurposable agents in addition to their intended targets. Although their study is the closest probe to ours available in the literature, Finan *et al.*^39^ do not fully link shortlisted targets to drug candidates, as they do not screen targets by the PD of their associated agents. The authors also do not consider the pragmatic considerations necessary for a drug repurposing project^39^; we address these concerns by considering only “Approved” small molecules, which are ideal candidates for repurposing. Finan *et al.* also state that target agonists and antagonists present differences in repurposablity; however, they do not apply this reasoning to further prioritize their target shortlist.^39^ We acknowledge that repurposablity is a function of target MOA by creating “target-action pairs,” shifting our analysis of repurposability away from target information alone and focusing more on the pharmacology of available agents that could be repurposed.

**Appendix Fig. A2.**
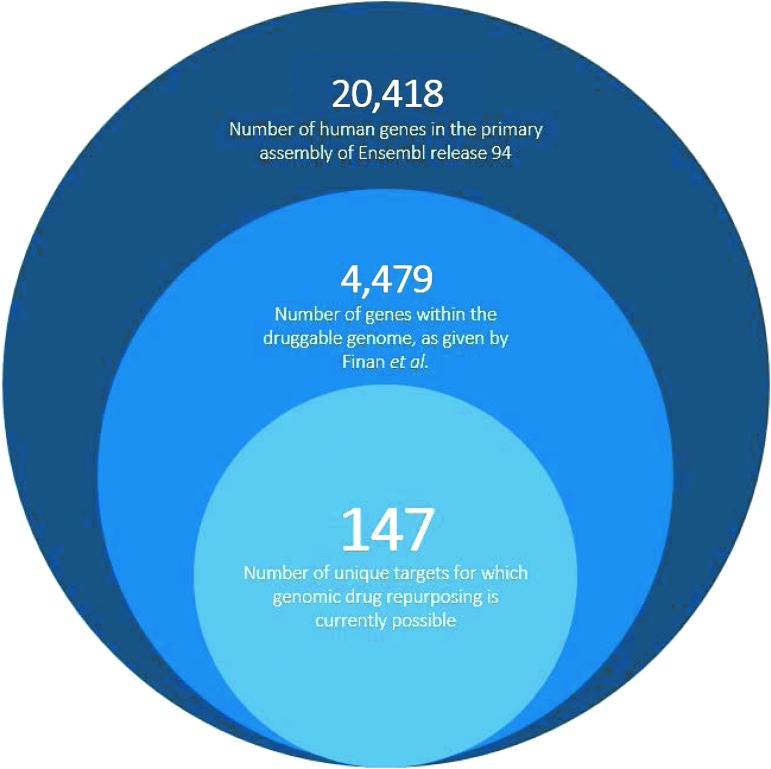
Our repurposability screens define the current scope of the genomic drug repurposing space (227 “target-action pairs” of 147 unique targets) within the entirety of the druggable genome.^A1,A2^

The comparison of our results with those of Finan *et al.*^39^ is given in *Appendix Figure A2*. We note that our procedure of removing “target-action pairs” with obvious and severe toxicity concerns is similar to the toxicity attrition scheme used in Finan *et al.*^39^

By comparing our approach to Finan *et al.*,^39^ we define the “repurposable drugged genome”: a new scope for the genomic repurposing space, consisting of 227 “target-action pairs.” We note that 147 targets (*Appendix Fig. A2*) within the druggable genome are associated with a repurposable, marketed small molecule. However, a large majority of targets within the druggable genome have not been harnessed for their *de novo* drug development potential.^41^ As new molecules for these targets are developed—and genomic association studies reveal significant SNVs within their associated genes—our definition of the scope of genomic drug repurposing will surely change.

## Conclusions and Recommendations for Future Study

Drug repurposing, as we approach it, is an interdisciplinary endeavor, benefitting from the integration of biomedical informatics, pharmacology, and genomics. We are most interested in using this approach to develop therapies for diseases with no available, effective treatments—regardless of the incidence of the disease or commercial potential. Our drug repurposing program, at present, has 3 clinical trials in progress (NCT03694249, NCT03617172, NCT03527472), included in the 14 total projects spread across 7 disease areas that we currently seek to address. Given that we establish the scope of all genomic drug repurposing, we observe that the workflow presented in this article is effective in reducing the necessary investment of time and money for lead identification in a drug repurposing RCT.

**Appendix Fig. A3.**
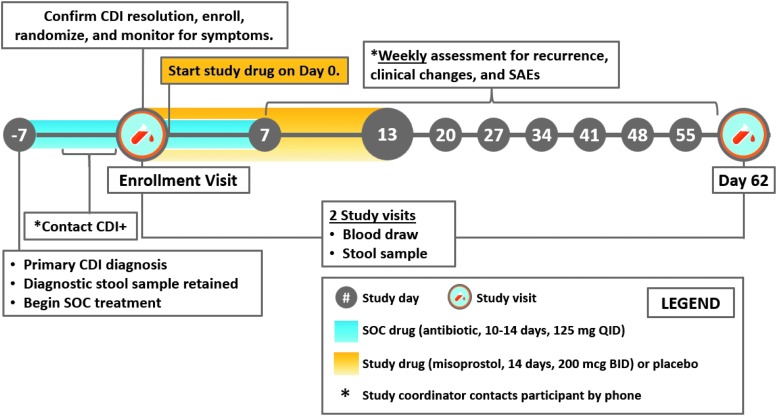
A summary of the trial design of NCT03617172, a phase II repurposing randomized controlled trial for the potency and efficacy of misoprostol to treat recurrent *Clostridioides difficile* infection.^A3^ BID, twice per day; CDI, *C. difficile* infection; QID, four times per day; SAE, serious adverse event; SOC, standard of care.

Indeed, the progress of our group toward NCT03617172 (“PROCLAIM—Prevent Recurrence of *Clostridium difficile* Infection with Misoprostol”) highlights the power of our approach to identify strong precision drug repurposing hits. This phase II RCT seeks to assess the safety and efficacy of misoprostol—originally indicated for the treatment of NSAID-induced ulceration and postpartum hemorrhage—in the prevention of recurrence of *C. difficile* infection in patients of at least 18 years of age during the first 8 weeks after completion of standard of care oral antibiotic therapy. A repurposing signal between misoprostol and an SNP on the type 2 prostaglandin E receptor (PTGER2) was detected by a workflow similar to that in this article; the associated PTGER2 PheWAS hits were significantly enriched for gastritis and duodenitis, and esophageal ulcer.^42^ More information on the design of NCT03617172 is available in *Appendix Figure A3*.

Overall, we propose an efficient data filtering and integration model, considering pharmacological data from a publicly available database and genomic data in a large-scale DNA repository. This allows for the entirety of the “drugged genome” to be reduced to a prioritized set of drugs that may be further considered for repurposing potential.

Future enhancement of this model will address the aforementioned limitations in drug screening. Enhanced consideration of a systematic attrition strategy for drug toxicity would be useful, requiring first the identification of an easily mineable variable available at a centralized location. This investigation provides a functional definition of repurposing within the “drugged genome.” Future work utilizing this study as a tool may explore PheWAS signals of shortlisted drugs generated from increasingly specified search criteria. Thus, this study provides a framework upon which later investigations can rely in determination of optimal repurposing candidates across the “drugged genome.”

We intend to use this framework in the continuing selection of drug repurposing candidates for our pipeline.

## Supplementary Material

Supplemental data

Supplemental data

## Abbreviations Used

ACE: angiotensin-converting enzyme
API: application programming interface
EHR: electronic health record
exmID: ExomeChip ID number
FDA: U.S. Food and Drug Administration
GWAS: genome-wide association study
MAF: minor allele frequency
MOA: mechanism of action
NCE: new chemical entity
NSAID: nonsteroidal anti-inflammatory drug
PD: pharmacodynamic
PheWAS: phenome-wide association study
RCT: randomized controlled trial
rsID: reference cluster ID number
SNP: single-nucleotide polymorphism
SNV: single nucleotide variant
SOC: standard of care
VUMC: Vanderbilt University Medical Center
XML: eXtensible Markup Language

## Appendix

**Appendix Table A1.** Drugs Not Withdrawn from the Clinic and Containing a DrugCard Listing “Approved” Were Maintained on the Shortlist of Repurposable Drugs

Maintained values of approval status	Eliminated values of approval status	
Approved	Investigational	Experimental
Approved/experimental	Vet_approved	Experimental/investigational
Approved/experimental/vet_approved	Withdrawn	Experimental/illicit/withdrawn
Approved/illicit	Nutraceutical	Experimental/vet_approved
Approved/illicit/investigational	Vet_approved/withdrawn	Approved/withdrawn
Approved/illicit/investigational/vet_approved	Investigational/withdrawn	Approved/vet_approved/withdrawn
Approved/investigational	Investigational/nutraceutical	Approved/illicit/investigational/withdrawn
Approved/illicit/vet_approved	Investigational/vet_approved	Approval/illicit/withdrawn
Approved/investigational	Illicit	Approved/nutraceutical/withdrawn
Approved/investigational/nutraceutical	Illicit/withdrawn	Approved/investigational/withdrawn
Approved/investigational/vet_approved	Illicit/vet_approved	Approved/investigational/vet_approved/withdrawn
Approved/nutraceutical	Illicit/investigational	Investigational/vet_approved/withdrawn
Approved/nutraceutical/vet_approved	Illicit/investigational/withdrawn	Illicit/investigational/vet_approved
	Experimental/illicit/investigational	Experimental/illicit

In contrast, drugs without this keyword—or those given as “Withdrawn” and “Approved”—were removed from consideration as nonrepurposable small molecules.

**Appendix Table A2.** A Selection of “Target-Action Pairs,” Each with MAF_W >0.001 and <5% Genotype Missingness for at Least One Single-Nucleotide Polymorphism/Single Nucleotide Variant from the ExomeChip

Target	Mechanism of action	Small molecule	Current indication(s)	Marketing start date
Muscarinic acetylcholine receptor M1	Antagonist	Dicyclomine	IBS, colicky abdominal pain, diverticulitis	November 5, 1950
	Antagonist	Cyclopentolate	Mydriasis and cycloplegia (for diagnostic purposes)	June 30, 1958
	Antagonist	Glycopyrronium	Salivary, tracheobronchial, and pharyngeal secretions, acid reflux, cardiac vagal inhibitory reflexes during induction of anesthesia and intubation, COPD	March 28, 1961
	Antagonist	Scopolamine	Colicky abdominal pain, bradycardia, sialorrhea, diverticulitis, IBS, motion sickness	January 1, 1966
	Antagonist	Clidinium	Peptic ulcer disease, colicky abdominal pain, diverticulitis, IBS	September 1, 1966
	Antagonist	Propantheline	Enuresis, hyperhidrosis, abdominal spasm, bladder spasm	December 14, 1981
	Antagonist	Pirenzepine	Peptic ulcer, gastric ulcer, and duodenal ulcer.	December 31, 1984
	Antagonist	Trihexyphenidyl	Parkinson's disease, extrapyramidal reactions	November 1, 1987
Tyrosine-protein kinase BTK	Inhibitor	Acalabrutinib	Mantle cell lymphoma	October 31, 2017
	Inhibitor	Ibrutinib	Mantle cell lymphoma, chronic lymphocytic leukemia, Waldenstrom's macroglobulinemia	November 7, 2013
Prostaglandin F2-alpha receptor	Agonist	Tafluprost	Elevated intraocular pressure	February 10, 2012
	Agonist	Travoprost		October 20, 2006
	Agonist	Latanoprost		March 20, 1995
	Agonist	Dinoprost tromethamine	Abortion of second-trimester pregnancy, induction of labor, vasodilation (for diagnostic purposes)	December 31, 1974
Alpha-1A adrenergic receptor	Agonist	Tetryzoline	Minor eye irritation	November 30, 1979
	Agonist	Methoxamine	Hypotension	December 31, 1950
	Agonist	Ergonovine	Postpartum hemorrhage, postabortion hemorrhage	December 31, 1939
Alpha-1A adrenergic receptor	Antagonist	Silodosin	BPH	March 23, 2009
	Antagonist	Tamsulosin		September 12, 1997
Heat-stable enterotoxin receptor	Agonist	Linaclotide	IBS with constipation, chronic idiopathic constipation	August 30, 2012
Toll-like receptor 7	Agonist	Imiquimod	Facial actinic keratosis, genital and perianal warts	February 25, 2010
P2Y purinoceptor 12	Antagonist	Prasugrel	Atherothrombosis, MI	July 10, 2009
	Antagonist	Ticlopidine	Thrombotic stroke	July 1, 1999
	Antagonist	Clopidogrel	Atherosclerosis	January 1, 1900
Substance-P receptor	Antagonist	Netupitant	Iatrogenic emesis	October 13, 2014
	Antagonist	Aprepitant		March 26, 2003
Estrogen receptor alpha	Ligand	Synthetic conjugated estrogens, A	Postmenopausal vulvar and vaginal atrophy, vasomotor atonia	May 12, 1999
	Ligand	Synthetic conjugated estrogens, B	Postmenopausal vulvar and vaginal atrophy, vasomotor atonia, vaginal dryness	April 24, 2006

Each row gives one “target-action” pair from the 227 “pairs” available for genomic drug repurposing, with its currently associated indications.^A4^

BPH, benign prostatic hyperplasia; BTK, Bruton's tyrosine kinase; COPD, chronic obstructive pulmonary disease; IBS, irritable bowel syndrome; MI, myocardial infarction.

**Appendix Table A3.** A Summary of the 14 Ongoing Repurposing Studies That Our Group Has Derived from Systematic Drug Repurposability Screening Analogous to That Presented in This Publication

Therapeutic area	Precision repurposed indication	Drug status	Developmental stage
Gastroenterology	Recurrent *Clostridium difficile* colitis	Generic	Currently enrolling for phase II RCT
	Ulcerative colitis	Reformulated generic	Preclinical/IND enabling
	Crohn's disease	Generic	RCT planned for 2019
Psychiatry/neurology	Chronic fatigue from elevated NE	Generic	Biomarker study currently enrolling
	Anti-NMDAR-associated NPSLE/lupus fog	Generic (higher dose)	Currently enrolling for phase II RCT
Oncology	Cancer metastasis	In development	Currently enrolling for pilot RCT
Neuropathy, pain, and inflammation	Trigeminal neuralgia	Reformulated generic	Enrolling for phase II RCT mid-2019
	Nerve-related headache pain	Reformulated generic	Preclinical/IND enabling
Nephrology/hematology	Hemolytic uremic syndrome	Generic	Preclinical/IND enabling
	Renal protection	Generic	Biomarker study planned for 2019
Skin, fibrotic and autoimmune disease	Wound healing in diabetic ulcer	Reformulated generic	Preclinical/IND enabling
	Sjögren's syndrome	Generic	Enrolling for phase II RCT in late 2019
	Sarcoidosis	Proprietary/generic	Preclinical
Pediatrics	Pediatric osteomyelitis	Reformulated generic	Preclinical/IND enabling

IND, Investigational New Drug; NE, norepinephrine; NMDAR, *N*-methyl-D-aspartate receptor; NPSLE, neuropsychiatric systemic lupus erythematosus; RCT, randomized controlled trial.
