# Supplementary material for: Systematically Prioritizing Candidates in Genome-Based Drug Repurposing
Source: Assay Drug Dev Technol. 2019 Dec 13;17(8):352–63. doi: 10.1089/adt.2019.950 (PMC6921094; doi:10.1089/adt.2019.950)
Supplement: Supplemental data [file Supp_Data1.pdf]

Supplemental Material

Systematically Prioritizing Candidates in Genome-Based Drug Repurposing

Anup P. Challa[1,*], Robert R. Lavieri[1], Judith T. Lewis[1], Nicole M. Zaleski[1], Jana K. Shirey-Rice[1], Paul A. Harris[2], Jill M. Pulley[1]

[1]Vanderbilt Institute for Clinical and Translational Research, Vanderbilt University Medical Center

[2]Department of Biomedical Informatics, Vanderbilt University Medical Center

[*]Corresponding author

Contact:

Anup P. Challa

Vanderbilt Institute for Clinical and Translational Research,

Vanderbilt University Medical Center,

2525 West End Avenue, Suite 600,

Nashville, Tennessee 38163,

United States of America

E-mail: anup.p.challa.1@vumc.org

Tel.: + 1-615-875-0085

Introduction

We provide this supplement to our manuscript "Systematically Prioritizing Candidates in Genome-Based Drug Repurposing" to provide the listing of drugs and "target-action pairs" which we evaluate in our protocol, as well as to present the results of each stage of filtration which we discuss within our methods. This supplement is organized by tab name on the accompanying Excel spreadsheet "drugData," which contains tabs with the drugs and/or "target-action pairs" maintained after application of the screen in the tab name to the previous listing of drugs. These tab names correspond directly to the filters that we describe in our manuscript.

Availability of Supplemental Data

The "drugData" spreadsheet is available at https://github.com/judytlewis/drugRepurposing/.

Filters

All Drug Candidates. The "rawData" tab of "drugData" contains all 10,505 drugs that were extracted from DrugBank 5.1.0 using the statistical software R and its XML and plyr packages. As consistent throughout similar literature, we define the space of all known drugs to be the collection of all DrugCards in DrugBank. Therefore, we take the tab "rawData" to contain all known drugs of interest, along with their associated approval and mechanism of action (MOA) information. Note that numerous fields within this tab were collapsed if they were not used explicitly in the subsequent analyses outlined in the Methods of our manuscript. A list of relevant variables is thus specified within the Methods section.

Small Molecules. The "smallMolOnly" tab of the spreadsheet contains all drugs of type "small molecule." As we discuss in our manuscript, we removed all drugs of type "biotech" from "rawData," given pragmatic concerns of repurposing biologic agents.

Approved Drugs. In “approvedOnly,” we maintain only drugs which explicitly contain the keyword “approved,” given the difficulty of repurposing non-approved compounds within the academic setting. Further discussion of our rationale for this filer may be found in the “Drugs by Approval Status” section of our Methods.

Drugs of One Target. In “byOneTargetMOAOnly,” we restrict the scope of our analysis to solely drugs of one (1) target of one (1) known MOA. Since a significant majority of drugs of one (1) target of known MOA fall within the set of drugs with only one (1) intended target, we assume coverage of drugs of only one known target in the remainder of our analysis.

“Target-Action Pairs”. Given that consider drugs of only one (1) target, we used the fields “target1” and “action1” to create “target-action pairs.” As described in the Methods, this was accomplished by organizing all drug listings with common values for “target1” and “action1” into clusters. In the “drugData” tab “groupedOneTargetMOA,” these clusters are color-coded by target: bordering rows highlighted in green give drug listings within a discrete “target-action pair.” If a “target-action pair” borders another “pair” highlighted in green, this cluster is colored yellow to distinguish it from the previous “pair.” Note that there is no minimum on the number of listings within a “target-action pair” (i.e., a “pair” may consist of one or many component drugs).

Drugs with SNP Coverage. As we describe in our Methods, this study involves the integration of SNP/SNV readouts from an Illumina Infinium HumanExome BeadChip array with the known “drugged genome.” To this end, we describe our rationale in maintaining only “target-action pairs” associated with targets for which there exists at least one SNP/SNV with MAF_W $\geq$ 0.001 and genotype missingness ≤ 0.05 on target genes. The “target-action pairs” that meet these

criteria are listed in the tab “bySNPSNVCoverage.”

Results and Final Remarks

Within the tab “finalPairs” in “drugData”, we present a cleaned version of the 227 “target-action pairs” that we believe represent all practical opportunities for genomic drug repurposing. To the information from the “bySNPSNVCoverage” tab, we append the marketing information that we describe in the Methods section of our manuscript to the listing of “target-action pars.” We intend to employ this information in further developing algorithms to identify repurposing leads on the basis of intellectual property availability.

Note that the “pairs” presented on this sheet represent attrition by the filters explained above, as well as reduction of our drug and “pair” shortlists by removal of repurposing candidates with known human safety concerns. We describe this *ad hoc* methodology in our manuscript and removed candidates posing safety concerns throughout each stage of the above filtration.

We hope that these drug listings aid our audience in their understanding of our procedure and allow them to easily replicate the results which we present in our manuscript. For additional data requests, please contact the Corresponding Author, whose contact information is provided above.
